# Advancements in cellular immunotherapy: overcoming resistance in lung and colorectal cancer

**DOI:** 10.3389/fimmu.2025.1554256

**Published:** 2025-02-05

**Authors:** Lijuan Qin, Yuan Li, Juan Liu, Xiaoqin An

**Affiliations:** ^1^ Department of Radiotherapy, Shanxi Province Cancer Hospital/Shanxi Hospital Affiliated to Cancer Hospital, Chinese Academy of Medical Sciences/Cancer Hospital Affiliated to Shanxi Medical University, Taiyuan, Shanxi, China; ^2^ Department of Respiratory Medicine, Shanxi Province Cancer Hospital/Shanxi Hospital Affiliated to Cancer Hospital, Chinese Academy of Medical Sciences/Cancer Hospital Affiliated to Shanxi Medical University, Taiyuan, Shanxi, China; ^3^ Department of Special needs Medicine, Shanxi Province Cancer Hospital/Shanxi Hospital Affiliated to Cancer Hospital, Chinese Academy of Medical Sciences/Cancer Hospital Affiliated to Shanxi Medical University, Taiyuan, Shanxi, China

**Keywords:** immunotherapy, CAR-T cells, lung cancer, colorectal cancer, resistance mechanisms

## Abstract

Immunotherapy has revolutionized cancer treatment, offering hope for patients with otherwise treatment-resistant tumors. Among the most promising approaches are cellular therapies, particularly chimeric antigen receptor T-cell (CAR-T) therapy, which has shown remarkable success in hematologic malignancies. However, the application of these therapies to solid tumors, such as lung and colorectal cancers, has faced significant challenges. Tumor resistance mechanisms—ranging from immune evasion, antigen loss, and immune checkpoint upregulation, to tumor microenvironment immunosuppression—remain major obstacles. This mini-review highlights the latest advancements in tumor immunotherapy, with a focus on cellular therapies, and addresses the resistance mechanisms that hinder their effectiveness in lung and colorectal cancers. We examine the evolution of CAR-T cell therapy, as well as the potential of engineered natural killer (NK) cells and macrophages in solid tumor treatment. The review also explores cutting-edge strategies aimed at overcoming resistance, including combination therapies, gene editing technologies, and nanotechnology for targeted drug delivery. By discussing the molecular, cellular, and microenvironmental factors contributing to resistance, we aim to provide a comprehensive overview of how these challenges can be overcome, paving the way for more effective, personalized immunotherapies in lung and colorectal cancer treatment.

## 1 Introduction

Immunotherapy has revolutionized cancer treatment, providing novel approaches that harness the body’s immune system to fight cancer. Among these, cellular immunotherapies, particularly chimeric antigen receptor T-cell (CAR-T) therapy, have shown remarkable success, particularly in hematologic malignancies. The ability to genetically modify T-cells to express receptors that recognize specific tumor antigens has enabled significant advances, leading to durable responses in cancers such as leukemia and lymphoma (1). However, the application of CAR-T therapy to solid tumors, including lung and colorectal cancers, has faced considerable challenges. Despite these challenges, recent innovations in cellular therapies have opened new avenues for overcoming resistance and improving the effectiveness of treatments for solid tumors.

Resistance to immunotherapies in solid tumors is multifactorial and presents a significant hurdle in the successful application of CAR-T therapy. The tumor microenvironment (TME) in solid cancers is often immunosuppressive, consisting of regulatory T cells (Tregs), myeloid-derived suppressor cells (MDSCs), and tumor-associated macrophages that inhibit immune responses (2). These immune cells, along with the presence of immunosuppressive factors like transforming growth factor-beta (TGF-β), create a hostile environment that limits the ability of T-cells to effectively target and kill tumor cells. Moreover, tumors often exhibit antigen loss or downregulation, which prevent CAR-T cells from recognizing their targets. Immune checkpoint molecules, such as PD-L1, are also upregulated in these tumors, allowing them to evade immune detection by inhibiting T-cell function (3).

Despite these challenges, significant strides have been made in enhancing cellular therapies to overcome the resistance mechanisms that hinder their effectiveness in solid tumors. Development of dual-CAR-T cell and the application of “off-the-shelf” cell therapies, such as allogeneic CAR-T cells or natural killer (NK) cells provide an alternative to autologous cell therapies, thereby improving accessibility and reducing the time required for treatment preparation. NK cell-based therapies are gaining traction due to the innate ability of NK cells to recognize and kill tumor cells without prior sensitization. NK cells can be genetically engineered to enhance their tumor-killing properties and, when combined with immune checkpoint inhibitors, have shown potential for overcoming the immunosuppressive TME. Similarly, macrophages, when reprogrammed into anti-tumor phenotypes, play a crucial role in targeting and eradicating solid tumors. These alternative cellular therapies offer promising strategies for addressing the limitations faced by CAR-T cells in treating lung and colorectal cancers.

This review explores the latest advancements in cellular immunotherapies, particularly CAR-T cells, NK cells, and macrophage-based therapies, with a focus on overcoming the resistance mechanisms in solid tumors. By understanding and addressing the unique challenges posed by lung and colorectal cancers, these emerging therapies offer new hope for improving the efficacy of immunotherapy and providing long-lasting responses for patients with advanced or resistant cancers.

## 2 Resistance mechanisms in tumor immunotherapy

**Figure 1:**
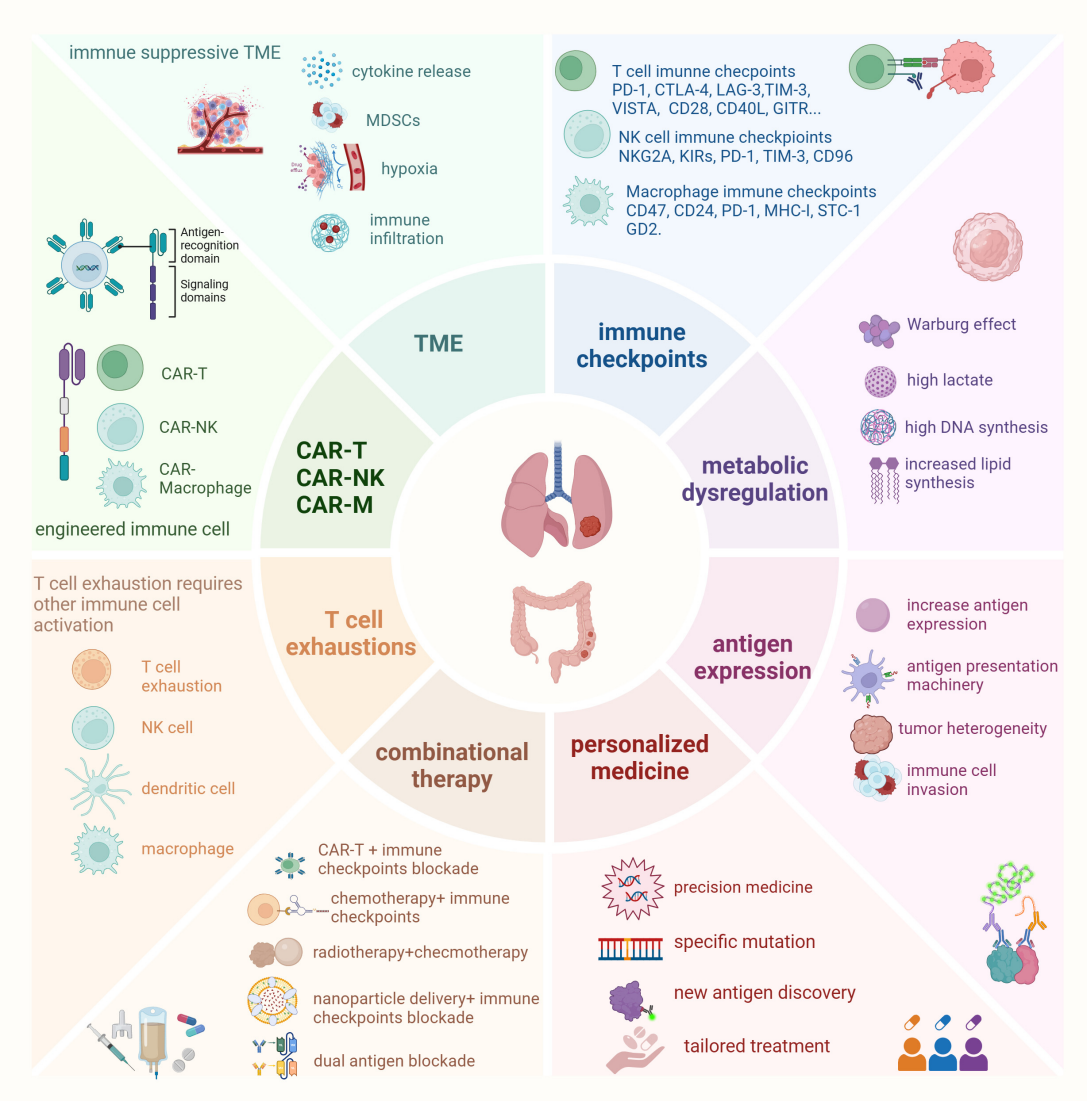
Main factors affect immune cell therapy in lung cancer and colorectal cancer Resistance to immunotherapy remains a significant challenge in lung cancer and colorectal cancer. Key factors contributing to the failure of immunotherapies include the tumor microenvironment (TME), immune escape mechanisms, and cellular dysfunctions. One emerging approach to overcome these challenges is targeting immune checkpoints on different immune cells, which has led to the development of new immunotherapies. In addition to CAR-T therapy, CAR-NK (chimeric antigen receptor natural killer cells) and CAR-Macrophages have garnered attention in preclinical research due to their specific advantages over CAR-T cells. However, T cell exhaustion and antigen loss represent new obstacles to cancer immunotherapy. Reversing T cell exhaustion requires not only the direct activation of T cells but also the activation of other immune cells, such as dendritic cells, NK cells, and macrophages, to modulate the overall immune response effectively. Moreover, metabolic dysfunctions in both immune cells and tumor cells play a critical role in affecting the efficacy of immune cell therapies. To address these challenges, combinational therapies and personalized medicine strategies are increasingly being encouraged. These approaches have shown promising results, offering hope for improving therapeutic outcomes in cancer immunotherapy.

Resistance to immunotherapy remains a significant challenge, particularly in solid tumors. The TME, immune escape mechanisms, and cellular dysfunctions are key factors contributing to the failure of immunotherapies (**Figure 1**). In this section, we will explore seven key resistance mechanisms that hinder the effectiveness of tumor immunotherapy, providing examples from both lung and colorectal cancers to illustrate how these mechanisms manifest in different tumor types.

### 2.1 Tumor microenvironment as a barrier to immunotherapy

TME plays a critical role in mediating resistance to immunotherapies (4). The TME is often characterized by factors that suppress immune activity and promote tumor survival. Hypoxia is a common feature of solid tumors, which leads to a lack of oxygen in the TME and induces the production of immunosuppressive cytokines (e.g., TGF-β, IL-10) and upregulation of immune checkpoint molecules (5). This hypoxic condition not only supports tumor growth but also impairs the function of immune cells. Additionally, the TME harbors a variety of immunosuppressive cell populations such as Tregs and MDSCs, which actively suppress the anti-tumor immune responses. These cells inhibit the activation of effector T-cells, promote tumor cell survival, and prevent immune cell infiltration into the tumor. The presence of these immune-suppressive cells and factors creates a protective niche for the tumor, hindering immune-based therapies to succeed.

The TME in non-small cell lung cancer (NSCLC) is often hypoxic, hypoxia promote cancer cell stemness and invasion by promoting glycolysis via lactylation of SOX9 (6), and it increased PD-L1 to suppress T-cell activation and function, Metabolic intervention that alleviates hypoxia and reduces PD-L1 expression enhances lung cancer radio-immunotherapy (7). Furthermore, NSCLC tumors are typically infiltrated with immunosuppressive cells like Tregs and MDSCs, which contribute to immune evasion by preventing effective anti-tumor immunity (8). High PD-L1 expression in NSCLC correlates with resistance to immune checkpoint inhibitors, such as pembrolizumab or nivolumab, highlighting the suppressive effects of the TME (9). In colorectal cancer (CRC), especially in microsatellite stable (MSS) tumors and metastatic tumors, the TME is also immunosuppressive (10). Tumors secrete TGF-β and IL-10, which promote immune suppression and contribute to therapy resistance. The presence of MDSCs and Tregs in CRC tumors prevents the activation of anti-tumor T-cells and inhibits the effectiveness of immunotherapies such as immune checkpoint inhibitors (11). MSS CRC tumors show poor response to anti-PD-1 therapy due to an immune-suppressive microenvironment, blocking IL-17A potentiates tumor response to anti-PD-1 immunotherapy in MSS CRC (12).

### 2.2 Antigen loss or downregulation

Tumor cells evade immune detection through antigen loss or downregulation. The genetic instability leads to the generation of new mutant antigens, many of which are immunogenic. However, tumors often adapt by downregulating or losing the expression of tumor-associated antigens (TAAs) or major histocompatibility complex (MHC) molecules, which are essential for the presentation of these antigens to immune cells. Loss of antigen expression enables the tumor to escape immune surveillance. In CAR-T therapy, where the engineered T-cells target a specific antigen on tumor cells, the loss of that antigen lead to therapeutic resistance, as the T-cells no longer recognize the tumor cells as targets (13). The alteration of surface antigens or clonal evolution in tumor cells complicates treatment, as therapies targeting a single antigen may become ineffective after these changes.

In NSCLC, tumor antigen loss is a significant issue, particularly for EGFR-targeted therapies. Tumors may lose or downregulate EGFR expression after prolonged treatment with EGFR tyrosine kinase inhibitors (TKIs) (14), leading to resistance. Additionally, PD-L1 downregulation hinders the effectiveness of checkpoint inhibitors. In NSCLC patients, the loss of HER2 or EGFR expression following targeted therapy leads to acquired resistance to treatments and targeting HER2 and EGFR exhibits positive immunotherapy results (15). CRC tumors with KRAS mutations or loss of MHC expression fail to present antigens effectively, reducing the ability of T-cells to recognize and attack tumor cells (16).

### 2.3 Immune checkpoint upregulation

Tumor cells use immune checkpoint molecules to inhibit immune function, effectively “braking” immune responses (17). Checkpoints like PD-1, CTLA-4, CD47 have been studied a lot in different cancers (18). In NSCLC, tumors often increase PD-L1 expression in response to inflammation, helping them evade immune detection by binding to PD-1 receptors on T-cells. Other checkpoint molecules, such as CTLA-4 and TIM-3, also contribute to resistance to PD-1/PD-L1 therapies. High PD-L1 levels in NSCLC are linked to resistance to PD-1/PD-L1 blockers, with relapse occurring after initial response. In CRC, especially MSS tumors, upregulated PD-L1 and CTLA-4 limit checkpoint inhibitor effectiveness. Even in microsatellite instability-high (MSI-H) CRC, resistance can arise if PD-L1 expression doesn’t increase, hindering an immune response.

### 2.4 T cell exhaustion and dysfunction

T-cell exhaustion is another critical mechanism of resistance to immunotherapy, particularly in chronic or persistent tumor environments. As T-cells persistently encounter tumor antigens in the TME, they undergo a process of functional decline, characterized by the upregulation of inhibitory receptors like PD-1, TIM-3, and LAG-3 (19). Exhausted T-cells exhibit reduced cytokine production, impaired proliferative capacity, and diminished cytotoxic function, making them less effective at killing tumor cells. This phenomenon is particularly pronounced in tumors that have a high degree of immune infiltration and antigen persistence. In advanced stages of NSCLC, T-cells often show exhaustion markers such as PD-1, TIM-3, and LAG-3, impairing their function (20). This is particularly problematic for patients treated with checkpoint inhibitors, as exhausted T-cells fail to mount an effective anti-tumor response. In NSCLC, patients treated with anti-PD-1 therapy who later relapse often exhibit increased PD-1 expression on T-cells, signaling exhaustion and reduced therapeutic efficacy. Similarly, in CRC, T-cell exhaustion is driven by chronic antigen exposure, particularly in tumors that are immune-infiltrated. The presence of exhausted T-cells in the TME contributes to resistance to therapies, including immune checkpoint inhibitors (21). T cell exhaustion in CRC is regulated by many infactors, cholesterol induces CD8^+^ T cell exhaustions by regulating endoplasmic reticulum-mitochondria contact (22); MGP promotes CD8^+^ T cell exhaustion by activating the NF-kB pathway and leading to cancer metastasis (23).

### 2.5 Metabolic dysregulation in tumors

The metabolic landscape of tumors plays a significant role in immune resistance. Tumor cells often undergo metabolic reprogramming, such as enhanced glycolysis (the Warburg effect), to support their rapid growth. This reprogramming not only promotes tumor progression but also affects immune cell function. Tumor metabolism in NSCLC often involves aerobic glycolysis, which not only provides energy for rapid tumor growth but also generates byproducts like lactate that acidify the TME. The Warburg effect enhanced by AKR1B10 promotes acquired resistance to pemetrexed in lung cancer-derived brain metastasis (24). High lactate level induces tumor-associated fibroblast activation and IL-8 mediated macrophage recruitment to potentiate lung cancer progression and compromise the immunotherapy (25). MCT-4-mediated lactate secretion inhibits antitumor immunity in LKB1-deficient lung cancer (26). This acidic environment suppresses T-cell function and promotes immune evasion. In addition, in lung adenocarcinoma, harnessing lipid metabolism modulation indicates improved immunotherapy outcomes (27). Mitochondrial networks and biogenetics also influence cancer cell immunotherapy in lung cancer (28). H3K18 lactylation enhances immune escape by activating the POM121/MYC/PD-L1 pathway in NSCLC (29). In CRC, lactate production from glycolysis and arginine depletion inhibit the function of effector T-cells, making tumors less responsive to immunotherapies. High lactate upregulates lactylation, the lactylation-driven METTL3-mediated RNA m6A modification promotes immunosuppression of tumor-infiltrating myeloid cells in colon cancer (30). Circulating L-arginine predicts the survival of cancer patients treated with immune checkpoint inhibitors in colon cancer (31). Moreover, the fusobacterium nucleatum-derived succinic acid induces tumor resistance to immunotherapy in colorectal cancer (32).

## 3 Approaches to overcoming immunotherapy resistance

Immunotherapy resistance remains a major obstacle in the effective treatment of cancers, especially in solid tumors like lung and colorectal cancer. Overcoming this resistance requires a multifaceted approach, involving a combination of strategies targeting different mechanisms of immune evasion. Key strategies include enhancing immune cell efficacy, overcoming immune suppressive factors in TME, and leveraging combination therapies.

### 3.1 Utilizing alternative cell therapies (NK cells and macrophages)

NK cells and macrophages are emerging as valuable alternatives to T-cell-based therapies like CAR-T (33, 34). NK cells target tumors without prior sensitization, they are less vulnerable to antigen loss and TME immunosuppression (35, 36). NK cell-based immunotherapies including combined cytokine, CDC and ADCC, NK-92, KIR mismatch and CAR approaches. CAR-NK cell therapy results in reduced toxicity, lower cost, and broader accessibility compared to CAR-T cells (37). Specifically, CAR-NK cells have a lower risk of graft-versus-host disease (GVHD), enabling “off-the-shelf” allogeneic therapies, and possess both CAR-mediated and innate antitumor activity, making them effective against heterogeneous tumors. They have a better safety profile with reduced risks of cytokine release syndrome (CRS) and neurotoxicity and a shorter lifespan, minimizing long-term side effects. Their scalability from various sources, like cord blood or iPSCs, further enhances accessibility. In a phase I/II trial, CD19 CAR-NK cells achieved a 73% response rate in patients with relapsed/refractory B-cell malignancies, without CRS or neurotoxicity (38). NK cells prime cancer cells for mt-apoptosis and combining them with BH3 mimetics enhances cancer cell death and tumor suppression. BH3 profiling helps identify the most effective mimetic, offering a precision strategy to improve NK-based and T cell-based immunotherapies (39). Rocaglamide enhances NK cell infiltration and antitumor immunity by activating the cGAS-STING signaling pathway in non-small cell lung cancer (40). NK-cell mediated therapy in lung cancer and CRC has been reviewed (41, 42).

Similarly, macrophages can be reprogrammed to exhibit anti-tumor properties, helping to eliminate resistant tumor cells and reprogram the TME for enhanced immune responses (43). CAR-macrophages have emerged as a new cancer immunotherapy to target solid tumors (44), offering several advantages over CAR-T cells. Unlike CAR-T cells, which rely primarily on direct cytotoxicity, CAR-macrophages utilize their natural phagocytic ability to engulf tumor cells and present tumor antigens, thereby activating downstream immune responses (45, 46). Their ability to infiltrate dense tumor microenvironments makes them particularly effective against solid tumors, a challenge for CAR-T therapies. The tandem CD3ζ-TIR dual signaling CAR design enables induced pluripotent stem cell-derived macrophages (iMACs) to engage targets, promote M1 polarization, resist immunosuppressive M2 polarization, and modulate the tumor microenvironment through an NF-κB-dependent mechanism (47). Furthermore, CAR-macrophages are less prone to exhaustion and can reprogram the immunosuppressive TME, enhancing the recruitment and activation of T cells and other immune effectors (44, 48). Preclinical studies of anti-HER2 CAR-macrophages demonstrated significant tumor growth inhibition in HER2^+^ xenograft models, emphasizing their ability to overcome antigen escape and address TME immunosuppression (49). These properties make CAR-macrophages a powerful alternative to CAR-T cells in tackling the unique challenges of solid tumors.

### 3.2 Improving antigen expression and new generation CAR-T design

Enhancing antigen targeting or using alternative antigens overcome this challenge. In NSCLC, novel antigen targets such as mesothelin or specific mutations like EGFR are being explored for CAR-T therapy, providing potential solutions to antigen loss and improving the targeting of resistant tumors (9, 50). Engineering CAR-T cells to target multiple antigens or incorporate novel features, such as cytokine secretion or checkpoint inhibition, could overcome issues of antigen loss. Moreover, IL-10-expressing CAR T cells maintain mitochondrial structure and function in the tumor microenvironment, IL-10 secretion boosted CAR T cell proliferation and effector functions, resulting in the complete regression of established solid tumors and metastatic cancers in multiple cancer types including colon in both syngeneic and xenograft mouse models (51). Dual-targeted CAR-T cells and “armored” CAR-T cells that resist the immunosuppressive TME represent exciting new avenues to enhance efficacy in solid tumors (52). In lung cancer, CAR-T cells with dual targets on HER2 and HLA-A02 enhance tumor specificity and address on-target off-tumor toxicity in HER2+ lung cancer cell lines with HLA-A02 loss of heterozygosity (53). Clinical trials of lung cancer targeting different antigens are listed in **Table 1**.

**Table 1:** Targeting antigens of lung cancer for immunotherapy registered in clinical trials.

NO.	Target	First posted time	Stage	Clinical trial ID number
1	PD-1 and TIL	10/2024	recruiting	NCT06538012
2	KK-LC-1	07/2024	recruiting	NCT05483491
3	GD2	10/2024	recruiting	NCT05620342
4	DLL3	02/2024	recruiting	NCT05680922
5	GPC3	12/2024	recruiting	NCT05120271
6	CEA,HLA-A*02	11/2024	recruiting	NCT05736731
7	MSLN	11/2024	recruiting	NCT06051695
8	MUCI	09/2024	recruiting	NCT05239143
9	GPC3	06/2024	recruiting	NCT06196294
10	EGFR/B7H3	06/2024	recruiting	NCT05341492
11	EGFR	06/2024	recruiting	NCT05060796
12	CEA	09/2023	recruiting	NCT06043466
13	GPC3	06/2024	recruiting	NCT03198546
14	CEA	11/2023	recruiting	NCT06126406
15	CEA	11/2023	recruiting	NCT06010862
16	CEA	08/2024	recruiting	NCT06006390
17	EGFR	10/2023	II	NCT05299125
18	PD-L1	06/2023	II	NCT05904015
19	CEA	04/2020	I/II	NCT04348643
20	CEA	01/2015	I	NCT02349724
21	CD276	04/2021	early phase I	NCT04864821
22	EGFR	11/2019	I	NCT05060796
23	EGFR	09/2021	early phase I	NCT05060796
24	HER2	11/2018	I	NCT03740256
25	HER2	12/2020	I	NCT04660929
26	HER2	09/2013	I/II	NCT01935843
27	MSLN	04/2012	I/II	NCT01583686
28	MSLN	02/2017	I	NCT03054298
29	MUCI	10/2015	I/II	NCT02587689
30	MUCI	05/2018	I/II	NCT03525782

CEA, carcinoembryonic antigen; EGFR, epidermal growth receptor; GPC3, glypican 3. MUCI, mucin 1; PD-L1, programmed death ligand 1; HER2, human epidermal growth factor 2; MSLN, mesothelin. TIL, tumor infiltrating lymphocytes.

### 3.3 Immune checkpoint Inhibition and CAR-T cell synergy

Tumor cells often exploit immune checkpoints such as PD-1/PD-L1, CTLA-4, and others to inhibit T-cell function. Combining checkpoint inhibitors with CAR-T therapy overcomes resistance by blocking these inhibitory signals, restoring immune cell function, and enhancing the anti-tumor response. Immunogenic chemotherapy enhances the recruitment of CAR-T cells to lung tumors, thereby improving the overall antitumor efficacy (54). When combined with checkpoint blockade therapy (anti-PD-1), this approach further boosts the therapeutic response, potentially leading to more effective treatment outcomes (55). In CRC, combining PD-1/PD-L1 inhibitors with CAR-T cells or NK cell therapy has demonstrated enhanced anti-tumor effects, particularly in tumors that express immune checkpoint molecules. The dual CAR-T cells with anti-PD-L1 scFv were capable of eradicating established tumors in mouse models of peritoneal metastasis of colorectal cancer (56).

Some of the advanced clinical trials have exhibited good results of chemotherapy in lung cancer. For instance, the phase III RATIONALE 303 trial (NCT03358875) demonstrated that Tislelizumab significantly extended overall survival in patients with advanced NSCLC who had progressed after platinum-based chemotherapy (57). Additionally, the ADRIATIC study showed that Duvatinib as a consolidation therapy after chemoradiotherapy significantly improved three-year survival rates in patients with limited-stage small cell lung cancer (SCLC) (58). However, for the cell therapies, they are still in the process.

### 3.4 Ameliorating immune cell exhaustion and senescence

T-cell exhaustion is a major cause of immune resistance (59). Immune checkpoint blockade has been used to prevent T-cell exhaustion, thereby enhancing CAR-T cell function. Additionally, NK cell therapy has shown potential to circumvent T-cell exhaustion, providing an alternative for overcoming resistance. Senescent T cells require NK cell to promote antitumor immunity (60). Combining anti-PD-1 inhibitors with CAR-T therapy or NK cells helps rejuvenate exhausted T cells and enhance immune responses against CRC. Additionally, improving the persistence of engineered immune cells is a focus of ongoing research in CRC. Clearance of senescent macrophages ameliorates tumorigenesis in KRAS-driven lung cancer (61).

### 3.5 Combination therapies: a multi-pronged approach

Combination therapies have emerged as one of the most effective strategies to overcome resistance to immunotherapy (62). By simultaneously targeting different immune pathways, combination approaches address the multiple layers of resistance mechanisms that tumors use to evade immune attacks. This multi-pronged strategy aims to enhance the overall efficacy of treatment and improve patient outcomes. In NSCLC, combinations of CAR-T cells, NK cells, and immune checkpoint inhibitors have demonstrated synergistic effects, enhancing anti-tumor activity (58). This approach is particularly valuable in addressing various resistance mechanisms, such as immune evasion and immunosuppression within TME. In CRC, combining CAR-T cells with immune checkpoint inhibitors or chemotherapy has shown promising potential to improve responses, particularly in patients who are resistant to monotherapies. For instance, the combination of nivolumab and ipilimumab, with or without chemotherapy, has been shown to offer long-term, durable clinical benefits in metastatic NSCLC patients with tumor PD-L1 expression below 1% (63). This combination supports its use as a first-line treatment option, especially in populations with high unmet needs. Furthermore, adding toripalimab to perioperative chemotherapy significantly improved event-free survival in patients with resectable stage III NSCLC, while maintaining a manageable safety profile (64). These findings underscore the potential of combination therapies to offer substantial clinical benefits by overcoming resistance mechanisms and providing lasting therapeutic effects in cancers.

### 3.6 Personalized and adaptive immunotherapies

Given the significant variability in immune responses across patients, personalized and adaptive immunotherapies are increasingly recognized as essential for optimizing cancer treatment (65). Precision medicine tailors treatments to the specific genetic and molecular characteristics of a patient’s cancer (66). Identifying tumor-specific neoantigens, which are unique to cancer cells and not present in normal tissues, can be used to develop vaccines or engineered immune cells, such as CAR-T,CAR-NK, CAR-Macrophage therapies (67). Moreover, tailoring therapies based on genetic and immune profiling has the potential to significantly enhance treatment outcomes. By analyzing both the genetic mutations of the tumor and the immune system’s response to it, specific targets will be discovered. This allows for more targeted therapies that minimize side effects while maximizing antitumor efficacy (68). For instance, lung cancer patients with specific genetic alterations, like EGFR mutations, may benefit from targeted therapies combined with immunotherapy, whereas others may respond better to conventional treatments or personalized CAR-T therapies. Ultimately, integrating these personalized and adaptive strategies into clinical practice will be crucial for improving the success of cellular therapies, especially in complex cancers like lung and colorectal cancer, where variability in patient responses is high.

Lung cancer and colorectal cancer exhibit distinct resistance mechanisms to immunotherapy and cell-based therapies due to differences in their tumor microenvironments and molecular characteristics. In lung cancer, particularly NSCLC, resistance often arises through the upregulation of immune checkpoint pathways like PD-1/PD-L1, which suppresses T-cell activity. Despite a high tumor mutational burden (TMB) in smokers’ lung cancers, resistance can still occur due to antigen loss or altered antigen presentation (9). Additionally, CAR-T therapies face resistance from poor T-cell infiltration or downregulation of target antigens like EGFR. In contrast, CRC often involves mismatch repair deficiency (dMMR) and microsatellite instability (MSI), leading to an elevated mutational burden. However, resistance in CRC is marked by immune exclusion, where T-cells fail to infiltrate the tumor. This is particularly evident in mismatch repair-proficient (pMMR) tumors. CRC also has an immunosuppressive tumor microenvironment, which limits the effectiveness of immunotherapies. Resistance to cell-based therapies in CRC arises from antigen heterogeneity and downregulation of target antigens. While both cancers share immune evasion and antigen loss mechanisms, lung cancer is more affected by checkpoint resistance and poor immune cell penetration, whereas CRC faces challenges with immune exclusion and an immunosuppressive microenvironment. These differences underscore the need for tailored treatment strategies.

## 4 Conclusion and perspective

Significant advancements have been made in the field of immunotherapy, particularly in the application of cellular therapies like CAR-T, NK cells, and macrophages for treating solid tumors, including lung and colorectal cancers. Despite the promising results, resistance to immunotherapies remains a major challenge. The complexity of TME, antigen escape, immune checkpoint overexpression, and T-cell exhaustion are key mechanisms of resistance. While CAR-T cell therapies have shown notable success in hematologic malignancies, their application in solid tumors like lung and colorectal cancers is still limited due to the unique obstacles posed by these cancers. Additionally, the development of alternative therapies, such as NK cell and macrophage-based approaches, offers exciting new avenues to overcome these barriers and broaden the scope of cellular immunotherapies.

Looking forward, a multi-pronged approach combining different immunotherapeutic strategies will likely be essential for overcoming resistance in solid tumors. Enhancing the efficacy of CAR-T cells through better tumor targeting, engineering of immune cells, and modulation of the TME holds significant promise. Moreover, leveraging combination therapies, which include immune checkpoint inhibitors, cytokines, and alternative cell-based therapies like NK cells and reprogrammed macrophages, may provide synergistic effects to overcome resistance mechanisms. Understanding the mechanisms of resistance at a molecular level and further optimizing therapeutic designs will be key to improving outcomes for lung and colorectal cancer patients. The future of cancer immunotherapy depends on combining these new approaches, which could transform treatment and greatly improve patient survival and quality of life.
